# Inadequate dietary diversity during pregnancy increases the risk of maternal anemia and low birth weight in Africa: A systematic review and meta‐analysis

**DOI:** 10.1002/fsn3.3388

**Published:** 2023-05-06

**Authors:** Awole Seid, Desta Dugassa Fufa, Misrak Weldeyohannes, Zuriyash Tadesse, Selamawit Lake Fenta, Zebenay Workneh Bitew, Getenet Dessie

**Affiliations:** ^1^ Department of Adult Health Nursing College of Medicine and Health Sciences, Bahir Dar University Bahir Dar Ethiopia; ^2^ Center for Food Sciences and Nutrition Addis Ababa University Addis Ababa Ethiopia; ^3^ Haramaya Institute of Technology, Haramaya University Dire Dawa Ethiopia; ^4^ Department of Anesthesia Addis Ababa University Addis Ababa Ethiopia; ^5^ Department of Nutrition and Dietetics Mekelle University Mekelle Ethiopia; ^6^ Department of Midwifery College of Medicine and Health Sciences, Bahir Dar University Bahir Dar Ethiopia; ^7^ St. Paul Hospital Millennium Medical College Addis Ababa Ethiopia; ^8^ National Center for Epidemiology and Population Health Australian National University, College of Health and Medicine Australian Capital Territory Canberra Australia

**Keywords:** Africa, anemia, dietary diversity, low birth weight, pregnancy, systematic review

## Abstract

Inadequately diversified food consumption during pregnancy can lead to micronutrient deficiencies, which can affect maternal and newborn health outcomes. Previous studies on maternal dietary diversity have either been limited to a specific geographical region or consist entirely of systematic reviews, without meta‐analyses. Thus, this study aimed to determine the pooled estimate of the association between inadequate dietary diversity during pregnancy, maternal anemia, and low birth weight in Africa. A systematic review of observational studies published between January 2000 and April 2022 was undertaken using the Google Scholar, PubMed, and CINAHL databases. The PRISMA checklist was followed to present the results. Microsoft Excel was used to abstract the data. STATA version 17 was used to analyze the data, and a random‐effects meta‐analysis model was applied to compute the pooled estimates. The study was registered in PROSPERO with protocol number CRD42022320873. A total of 22 publications with 9,696 participants were included in the final meta‐analysis. The pooled adjusted odds ratio (AOR) for inadequate dietary diversity and maternal anemia was 2.15 (95% CI, 1.66–2.65), while that for low birth weight was 2.04 (95% CI, 1.46–2.63). The highest pooled estimate of maternal anemia was reported in Cameroon (AOR = 9.8, 95% CI: 1.68–17.92), followed by Ethiopia (AOR = 2.6, 95% CI: 1.95–3.25). Similarly, the pooled estimates of low birth weight were highest in Cameroon (AOR = 3.04, 95% CI: 1.19–4.88) and Ethiopia (AOR = 1.8, 95% CI: 1.29–2.39). In Africa, pregnant mothers with inadequate dietary diversity are two times more likely to develop anemia and low birth weight. Social protection policies that prioritize pregnant women, maternal nutrition promotion in the community, and dietary counseling during antenatal care visits, using national food‐based dietary guidelines, should be strengthened.

## Graphical abstract

Malnutrition among pregnant women in Africa remains high. In Africa, a lack of dietary variety during pregnancy doubles the odds of developing maternal anemia and low birth weight. Pregnant women should receive adequate dietary diversity counseling during prenatal visits. Micronutrient supplementation for pregnant women should be improved in places where food access is a barrier.

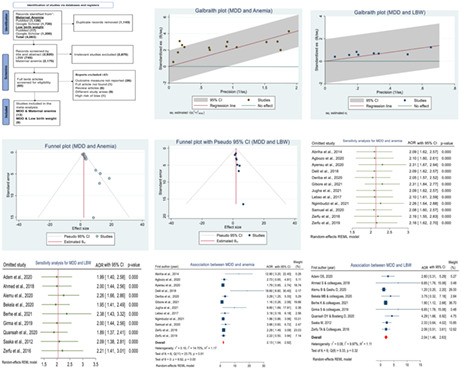

## 1 INTRODUCTION

One of the 13 goals for Sustainable Development Goal 3 (SDG‐3) on health agreed upon the international community in 2015 was to improve maternal health (WHO, 2019a). Suboptimal maternal nutrition during pregnancy is a common problem in developing nations and is associated with adverse health outcomes for both mothers and children (Tran et al., 2019). A meta‐analysis revealed that malnutrition among pregnant women in Africa reached 23.5% in 2019 (Hidru et al., 2020) and a study in Ghana demonstrated that the risk of malnutrition was higher in the late trimester (Saaka et al., 2017). Similarly, the prevalence of maternal anemia in different regions of Africa is 35.6% in sub‐Saharan Africa (Fite et al., 2021), 41.82% in East Africa (Liyew et al., 2021), 31% in South Africa (Dorsamy et al., 2022), 31.6%–53% in Ethiopia (Hidru et al., 2020; Kassa et al., 2017), and 53% in Sudan (Adam et al., 2018). It has been shown that during pregnancy, women with severe anemia are twice as likely to die during or shortly after pregnancy as women without anemia (UNICEF, 2022). Iron and folate deficiency anemia (IDA) during pregnancy can be prevented or controlled by iron supplementation, fortification, and improved dietary diversity (Da Silva Lopes et al., 2021).

Inadequate nutrition before and during pregnancy is an underlying cause of poor birth outcomes such as small for gestational age (SGA) and low birth weight (LBW) (Christian et al., 2013; WHO, 2019b). LBW (less than 2500 g at birth) is a global public health problem in low‐ and middle‐income countries and is potentially associated with wasting and stunting (Kim & Saada, 2013). It has been indicated that improving maternal nutrition is one component of a comprehensive global strategy to reduce the incidence of LBW (da Silva Lopes et al., 2017). Furthermore, a study found that 15%–20% of all births worldwide are LBW, amounting to more than 20 million births per year. Regional estimates of low birth weight are 28% in South Asia, 13% in sub‐Saharan Africa, 10%–15.7% in five African countries, and 9% in Latin America (He et al., 2018). Recognizing the critical role of maternal nutrition, the World Health Assembly has set targets to reduce the prevalence of anemia in women of reproductive age by 50% and that of low birth weight by 30% by 2025 (UNICEF, 2021; WHO, 2014).

Dietary diversity is an indicator of diet quality and is measured as the number of individual food items or food groups consumed over a given reference period (Kennedy et al., 2010). Furthermore, the Minimum Dietary Diversity for Women (MDD‐W) is a dichotomous indicator that women aged 15–49 consume at least five of the 10 food groups. The 10 food groups were grains, white roots, tubers, plantains, eggs, legumes (beans, peas, and lentils); dark green, leafy vegetables; nuts and seeds; other vitamin‐A‐rich fruits and vegetables; dairy products and milk; other vegetables; meat, poultry, and fish; and other fruits (FAO, 2021). According to this methodology, women who achieve the least dietary diversity are supposed to have good food access and dietary quality and meet their micronutrient intake recommendations, such as vitamins A, D, E, folate, calcium, iron, and magnesium (Aboagye et al., 2021; Gómez et al., 2020). Evidence shows that low dietary diversity during pregnancy is a valuable predictor of maternal anemia and LBW infants (Geta et al., 2022; Kheirouri & Alizadeh, 2021; Quansah & Boateng, 2020; Samuel et al., 2020).

Despite this, the consumption of invariant and monotonous diets (inadequate dietary diversity) is especially severe among poor populations in developing countries such as Africa (Azene et al., 2021). A meta‐analysis study in Ethiopia found that 56.6% of pregnant women and 50.21% of lactating women had inadequate dietary diversity and were below the FAO recommendations (Bitew et al., 2021). Indeed, it is an opportunity that countries in Africa (Benin, Ethiopia, Nigeria, Ghana, Kenya, Namibia, South Africa, Zambia, Seychelles, and Sierra Leone) have developed national food‐based dietary guidelines (FBDG) that serve as a guide for dietary counseling, and diet diversification was promoted as the most common recommendation across African country FBDGs (Ainuson‐Quampah et al., 2022).

Furthermore, governments and nongovernmental organizations, such as Nutrition International (NI), have been working in African countries (Ethiopia, Kenya, Tanzania, Nigeria, and Senegal) to improve maternal and child nutrition services. Some of the programs implemented in Africa include iron and folic acid (IFA) supplementation, promotion of food fortification, introduction of double‐fortified salt (iodine and folate) into the market, and improving the intake of essential vitamins and minerals to reduce the prevalence of anemia among pregnant and lactating women (Nutrition International, 2022). Furthermore, the WHO recommends (2020 update) daily multiple micronutrient supplements (MMS) with iron and folic acid in settings with poor dietary quality to improve micronutrient intake during pregnancy, prevent maternal anemia, and reduce low birth weight (WHO, 2020). Despite all the existing efforts, maternal anemia and low birth weight remain significant problems in low‐ and middle‐income countries (Figueiredo et al., 2018; Marete et al., 2020; Tessema et al., 2021).

On the other hand, evidence has shown that supplementation‐ and fortification‐based strategies are shortcuts to addressing the widely prevalent micronutrient deficiencies (MNDs). For example, a study in India highlighted food‐based approaches, such as dietary improvement and diversification as a long‐term and sustainable strategy to control MNDs and promote healthier lives (Chaudhary et al., 2022). However, in Africa, government efforts to promote dietary diversity have not been widely observed. Therefore, this study may contribute to the promotion of dietary diversity by showing the odds of maternal anemia and low birth weight attributed to inadequate maternal dietary diversity.

Previous systematic reviews have pointed out several associations between maternal dietary diversity, anemia, and low birth weight (Kheirouri & Alizadeh, 2021). However, a closer examination of these reviews reveals some limitations. First, they merely described the presence of associations in individual studies (narrative synthesis), without a quantitative summary of the strength of the association. Second, despite the existing evidence on the association between maternal dietary diversity and geographical and socioeconomic variations among countries, subgroup analysis at the regional (Africa) level has not been reported (Gete et al., 2020). In addition, several new observational studies with inconsistent results, not included in previous reviews, have emerged in recent years (Adem et al., 2020; Berhe et al., 2021; Girma et al., 2019). Finally, we examined an additional outcome variable, maternal anemia, which correlates with dietary diversity and low birth weight (Figueiredo et al., 2018; Rahman et al., 2016; Rahmati et al., 2017).

Therefore, in order to fill this gap, this study aimed to carry out a meta‐analysis by including all relevant observational studies available thus far, and to estimate the strength of the association between dietary diversity during pregnancy and maternal anemia and low birth weight in Africa. This will improve the precision of the estimated odds ratio by examining heterogeneity, publication bias, and sensitivity analysis to produce an average effect estimate representative of the African region. Notably, this will help policymakers to design policies that improve maternal dietary diversity in parallel with other public health interventions to prevent maternal anemia and LBW in Africa, where many of the countries are developing.

Before searching for articles, the scope of the study and eligibility criteria were defined using the Population, Intervention, Comparison, Outcomes, and Study (PICOS) framework (Amir‐Behghadami & Janati, 2020).

- Population (P): Pregnant women and newborns
- Intervention or exposure (I): Inadequate dietary diversity
- Comparison (C): Adequate dietary diversity
- Outcome (O): Maternal anemia (primary outcome), low birth weight (secondary outcome)
- Study (S): Observational studies (cross‐sectional, case–control, and cohort studies).
- Time frame (T): 2000–2022

### 1.1 Review question?

1. How strong is the association between inadequate dietary diversity during pregnancy and the risk of maternal anemia in Africa?
2. What is the strength of the association between inadequate dietary diversity during pregnancy and the risk of low birth weight in Africa?

## 2 METHODS

This systematic review and meta‐analysis were conducted following the Preferred Reporting Items for Systematic Reviews and Meta‐Analyses Checklist (PRISMA 2020 Statement) (Page et al., 2021).

### 2.1 Searching strategy and study selection

A systematic search of PubMed, Google Scholar, and Cochrane Library was performed using keywords and medical subject headings (MeSH). Additional relevant articles were identified using snowball sampling. Articles published from February 2000 to April 2022 were included, and the search was limited to studies in Africa and English. The article search ended on April 7, 2022. The selected studies were managed using EndNote version 20. The following terms were used as keywords and MeSH terms in PubMed and Google Scholar (Table 1).

**TABLE 1:** Search terms used to access articles in PubMed and Google Scholar databases, 2022.

Outcome variable	PubMed	Google Scholar
Maternal anemia	(((((“Diet”[MeSH Terms] OR “Diet”[All Fields] OR “dietary”[All Fields] OR “dietaries”[All Fields]) AND (“diverse”[All Fields] OR “diversely”[All Fields] OR “diversities”[All Fields] OR “diversity”[All Fields])) OR ((“Diet”[MeSH Terms] OR “Diet”[All Fields] OR “dietary”[All Fields] OR “dietaries”[All Fields]) AND (“varieties”[All Fields] OR “variety”[All Fields])) OR ((“food”[MeSH Terms] OR “food”[All Fields]) AND (“diverse”[All Fields] OR “diversely”[All Fields] OR “diversities”[All Fields] OR “diversity”[All Fields])) OR ((“food”[MeSH Terms] OR “food”[All Fields]) AND (“varieties”[All Fields] OR “variety”[All Fields])) OR “Diet”[MeSH Terms] OR “diet, healthy”[MeSH Terms]) AND “Pregnancy”[MeSH Terms] AND “pregnancy complications, hematologic”[MeSH Terms]) OR ((“maternally”[All Fields] OR “maternities”[All Fields] OR “maternity”[All Fields] OR “mothers”[MeSH Terms] OR “mothers”[All Fields] OR “maternal”[All Fields]) AND (“anaemia”[All Fields] OR “anemia”[MeSH Terms] OR “anemia”[All Fields] OR “anaemias”[All Fields] OR “anemias”[All Fields]))) AND 2000/01/01:2022/12/31[Date ‐ Publication] AND “Africa”[MeSH Terms]	‘maternal’ OR ‘pregnancy’ AND ‘dietary diversity’ OR ‘diet diversity’ OR ‘food diversity’ OR ‘dietary variety’ OR ‘diet variety’ OR ‘food variety’ AND ‘anemia’ with at least one of the words: ‘dietary diversity’ OR ‘diet diversity’ OR ‘food diversity’ OR ‘dietary variety’ OR ‘diet variety’ OR ‘food variety’ with the exact phrase: anemia
Low birth weight	(((“Diet”[MeSH Terms] OR “Diet”[All Fields] OR “dietary”[All Fields] OR “dietaries”[All Fields]) AND (“diverse”[All Fields] OR “diversely”[All Fields] OR “diversities”[All Fields] OR “diversity”[All Fields])) OR ((“Diet”[MeSH Terms] OR “Diet”[All Fields] OR “dietary”[All Fields] OR “dietaries”[All Fields]) AND (“varieties”[All Fields] OR “variety”[All Fields])) OR ((“food”[MeSH Terms] OR “food”[All Fields]) AND (“diverse”[All Fields] OR “diversely”[All Fields] OR “diversities”[All Fields] OR “diversity”[All Fields])) OR ((“food”[MeSH Terms] OR “food”[All Fields]) AND (“varieties”[All Fields] OR “variety”[All Fields])) OR “Diet”[MeSH Terms] OR “diet, healthy”[MeSH Terms]) AND “Pregnancy”[MeSH Terms] AND “infant, low birth weight”[MeSH Terms] AND “Africa”[MeSH Terms]	‘dietary diversity’ OR ‘diet diversity’ OR ‘food diversity’ OR ‘dietary variety’ OR ‘diet variety’ OR ‘food variety’ AND ‘low birth weight’ with at least one of the words: ‘dietary diversity’ OR ‘diet diversity’ OR ‘food diversity’ OR ‘dietary variety’ OR ‘diet variety’ OR ‘food variety’ with the exact phrase: low birth weight

#### 2.1.1 Eligibility criteria

Observational studies (cross‐sectional, case–control, and cohort) addressing the relationship between maternal dietary diversity and the risk of anemia in mothers and LBW in newborns published since 2000 in English and African countries, were eligible for analysis. Moreover, the dietary diversity reported in nonpregnant women, related to anemia in children, randomized controlled trials, and reviews were excluded. The initial year (2000) was selected because organizations such as UNICEF evaluated their programs by looking at the trend of maternal undernutrition, anemia, and low birth weight starting from 2000 onwards (WHO, 2019b). This meta‐analysis result will support efforts toward evidence‐based policymaking regarding maternal and child undernutrition. Moreover, widening the year interval will increase the number of articles included in the meta‐analysis, leading to a more precise estimate.

#### 2.1.2 Study selection

Eligible articles were transferred to EndNote 20 and duplicate articles were removed. The titles, abstracts, and full texts of eligible articles were critically reviewed independently by two authors (AS and ZWB) before proceeding with data extraction. Studies reporting different effect estimates and unmatched reference categories were excluded. Discrepancies in inclusion were resolved through discussions.

#### 2.1.3 Outcome interests

The primary and secondary outcome variables were maternal anemia and low birth weight. Anemia was defined as a serum hemoglobin level of less than 11 mg/dL, and low birth weight was defined as a birth weight of less than 2500 g (WHO, 2014). Dietary diversity was measured using the Food and Agriculture Organization MDD‐W tool. Pregnant mothers who consumed <5 of the 10 food groups were considered inadequate (FAO, 2021).

#### 2.1.4 Data extraction

All authors were involved in the data extraction process, which was performed using Microsoft Excel. The extracted data template was as follows: first author, year of publication, country, study design, sample size, study setting, method of dietary diversity assessment, number of days assessed, number of food groups considered, and cutoff point for inadequate dietary diversity. Statistical measures such as the adjusted odds ratio, confidence interval, and standard errors of the adjusted odds ratio were also extracted.

#### 2.1.5 Quality assessment

Two independent reviewers (AS and ZWB) examined the methodological qualities of the selected studies. During the selection process, disagreements between the authors were solved through discussion with a third author (MW). The Newcastle–Ottawa Scale (NOS) quality assessment tool for cohort, case–control, and cross‐sectional studies was used to evaluate the quality and risk of bias. Selection, comparability, and outcome are the three domains of NOS. High‐quality studies with a total score ≥6 were included in the meta‐analysis.

#### 2.1.6 Data processing and statistical analysis

After data were abstracted using Microsoft Excel, they were exported to Stata version 17 for further analysis. Heterogeneity across studies was assessed using the Chi‐squared (*I* ^2^) statistic, variance (*τ* ^2^), standard deviation (*τ*), and prediction interval. This was also assessed using a Galbraith plot. A visual funnel plot and Egger's test statistics were used to examine publication bias. A random‐effects model was used to estimate the pooled adjusted odds ratios (ORs). One study highlighted that the decision to use a meta‐analysis model should not be based only on statistical heterogeneity, although this study resulted in a heterogeneity test of *I* ^2^ = 14.79% for maternal anemia and 9.97% for low birth weight. Therefore, a random‐effects model was selected for the following reasons: the study intended to generalize the results to Africa beyond the included studies or countries; the number of articles included was more than five for both outcome variables; dietary patterns are usually affected by geographical settings and socioeconomic factors; the studies included used different dietary assessment methods; and there was an uneven cutoff point for classifying the exposure variable, that is, maternal dietary diversity (Tufanaru et al., 2015).

## 3 RESULTS

### 3.1 Study selection

**FIGURE 1:**
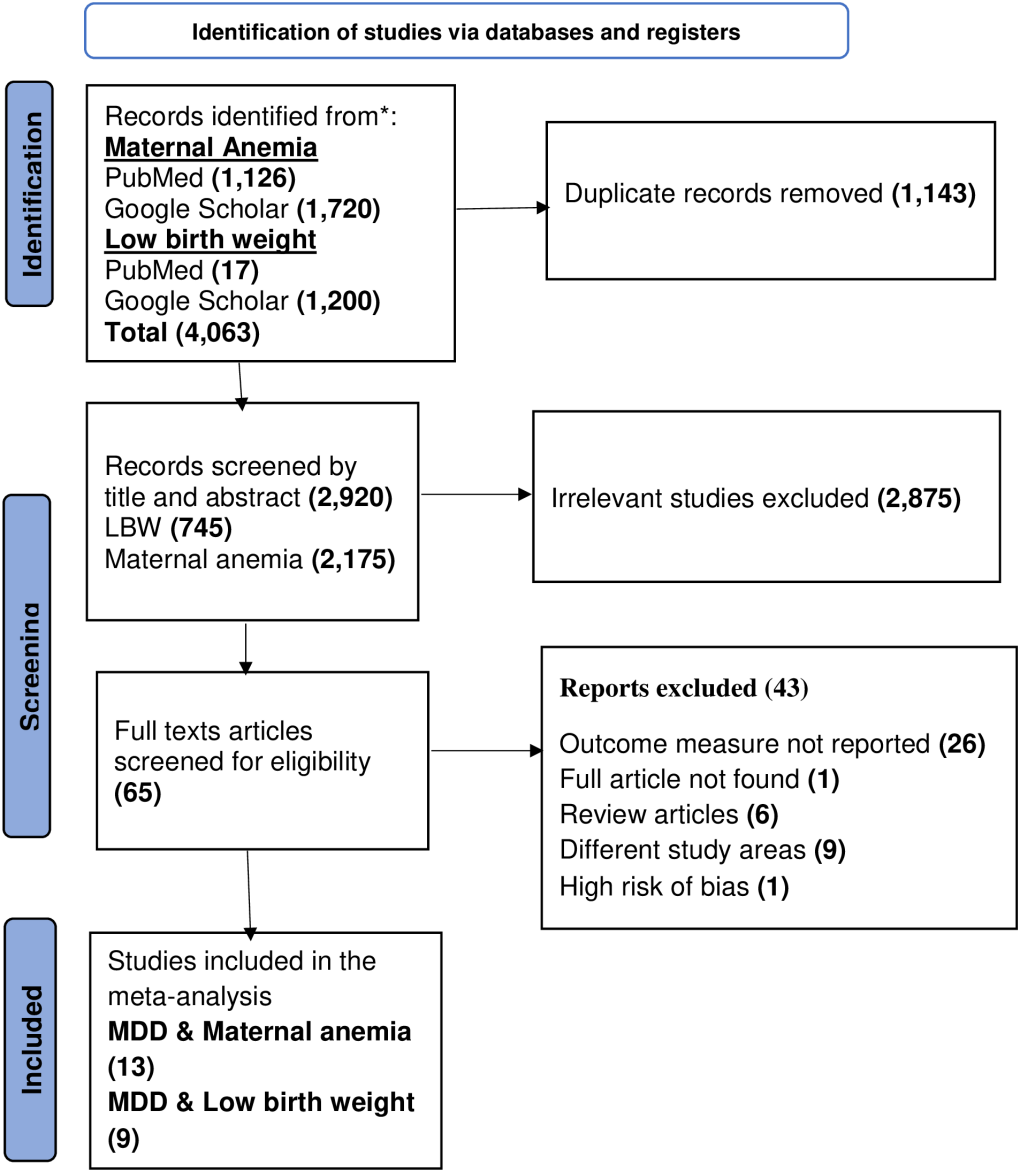
PRISMA flow chart diagram that shows the study selection process included in the meta‐analysis, 2022.

This review had two outcomes: maternal anemia and low birth weight. We searched 1143 PubMed publications and 2920 Google Scholar articles to determine the association between dietary diversity, maternal anemia, and low birth weight. After 1143 duplicates were removed from EndNote, 2920 records were screened by reading the titles and abstracts. Of these, 65 studies met the criteria for a full‐text review. After a full‐text review, 42 studies were excluded for several reasons, including lack of full text, being outside the scope of the study, not presenting outcome measures, having different reference categories, and a high risk of bias. After a thorough review, 13 articles for the association between inadequate dietary diversity and anemia (Abriha et al., 2014; Agbozo et al., 2020; Ayensu et al., 2020; Delil et al., 2018; Deriba et al., 2020; Gibore et al., 2021; Jugha et al., 2021; Lebso et al., 2017; Ngimbudzi et al., 2021; Samuel et al., 2020; Vanié et al., 2021; Zerfu et al., 2016, 2019) and nine articles for determining the strength of the association between inadequate dietary diversity and low birth weight (Adem et al., 2020; Ahmed et al., 2018; Alemu & Gashu, 2020; Bekela et al., 2020; Berhe et al., 2021; Girma et al., 2019; Quansah & Boateng, 2020; Saaka et al., 2017; Zerfu et al., 2016) were included in the final meta‐analysis (Figure 1). One study was discarded after quality assessment (Vanié et al., 2021).

### 3.2 Study characteristics

Of the 13 studies included to determine the association between dietary diversity and anemia, eight were reported from Ethiopia, two from Ghana, two from Tanzania, and one from Cameroon. The studies were published between 2014 and 2021. The sample sizes of the included studies ranged from 314 to 1014. Regarding the study design, nine articles were cross‐sectional, three were prospective cohort studies, and the rest were case–control studies. Furthermore, 12 studies used the FAO MDD‐W scale to measure women's dietary diversity scores, but the cutoff point for “inadequate dietary diversity” was inconsistent (Table 2).

**TABLE 2:** Study characteristics of inadequate dietary diversity during pregnancy and maternal anemia in Africa, 2022.

Author (s), year, ref	Country	Study design	Sample size	Study location	Dietary assessment method	Duration of food intake	Cutoff for inadequate DD	AOR	NOS score
Abriha et al., 2014	Ethiopia	Cross‐sectional	619	Health facilities	Not specified	Not specified	Not specified	12.8	7
Agbozo et al., 2020	Ghana	Prospective cohort	415	Health facilities	FFQ	1 week	<5/10	2.73	6
Ayensu et al., 2020	Ghana	Cross‐sectional	379	Community	24‐h recall	3 days	<5/10	1.795	8
Delil et al., 2018	Ethiopia	Cross‐sectional	314	Health facilities	FFQ	1 week	<6/10	18.6	10
Deriba et al., 2020	Ethiopia	Case–control	426	Health facilities	24‐h recall	1 day	≤3/9	3.29	7
Gibore et al., 2021	Tanzania	Cross‐sectional	338	Health facilities	24‐h recall	1 day	≤4/9	1.16	6
Jugha et al., 2021	Cameroon	Cross‐sectional	1014	Hospital	24‐h recall	1 day	<5/10	9.8	9
Lebso et al., 2017	Ethiopia	Cross‐sectional	507	Community	24‐h recall	1 day	<5/10	3.18	10
Ngimbudzi et al., 2021	Tanzania	Cross‐sectional	418	Hospital	24‐h recall	1 day	≤5/10	1.96	8
Samuel et al., 2020	Ethiopia	Cross‐sectional	423	Health facilities	24‐h recall	1 day	≤4/9	3.66	8
Tulu et al., 2019	Ethiopia	Case–control	573	Health facilities	Not specified	Not specified	Not specified	12.3	6
Vanié et al., 2021	Côte d'Ivoire	Cross‐sectional	389	Health facilities	24‐h recall	1 day	≤3/9	8.35	5
Zerfu et al., 2016	Ethiopia	Prospective cohort	374	Health Facilities	24‐h recall	1 day	<4/10	2.29	8
Zerfu et al., 2019	Ethiopia	Prospective cohort	432	Health facilities	24‐h recall	1 day	<5/10	2.22	8

Furthermore, regarding the association between maternal dietary diversity and low birth weight, nine articles and 3464 participants were included, of which seven were from Ethiopia and two were from Ghana. Sample sizes of included studies ranged from 279 to 540. Four studies were case–control, three were cohort studies, and the rest were cross‐sectional studies. A food frequency questionnaire (FFQ) and 24‐h dietary recall were used to assess dietary intake (Table 3).

**TABLE 3:** Study characteristics of inadequate dietary diversity during pregnancy and low birth weight in Africa, 2022.

Author (s), year, ref	Country	Study design	Sample size	Dietary assessment method	Duration of food intake	Cutoff for inadequate DD	AOR	NOS score
Adem et al., 2020	Ethiopia	Case–control	464	FFQ + 24‐h	7 days	≤3/9	2.8	7
Ahmed et al., 2018	Ethiopia	Case–control	286	24‐h recall	1 day	<5/10	6.65	7
Alemu and Gashu, 2020	Ethiopia	Prospective cohort	223	24‐h recall	1 day	≤5/10	1.2	7
Bekela et al., 2020	Ethiopia	Case–control	354	24‐h recall	1 day	<5/10	3.75	7
Berhe et al., 2021	Ethiopia	Prospective cohort	540	Not specified	Not specified	<5/10	1.9	8
Girma et al., 2019	Ethiopia	Case–control	279	24‐h recall	1 day	<5/10	6.65	7
Quansah and Boateng, 2020	Ghana	Cross‐sectional	420	FFQ	1 week	≤5/10	4.29	6
Saaka et al., 2017	Ghana	Cross‐sectional	524	FFQ	7 days	<8	2.33	7
Zerfu et al., 2016	Ethiopia	Prospective cohort	374	24‐h recall	1 day	<4	2.06	8

### 3.3 Quality of articles

One of the included studies was of poor quality based on NOS measurements and was, therefore, discarded from the analysis. Quality assessment was performed for each study design. Cross‐sectional studies had a quality score of 6–10, case–control studies had a score of 6–7, and prospective cohort studies had a score of 6–8.

### 3.4 Heterogeneity test

**FIGURE 2:**
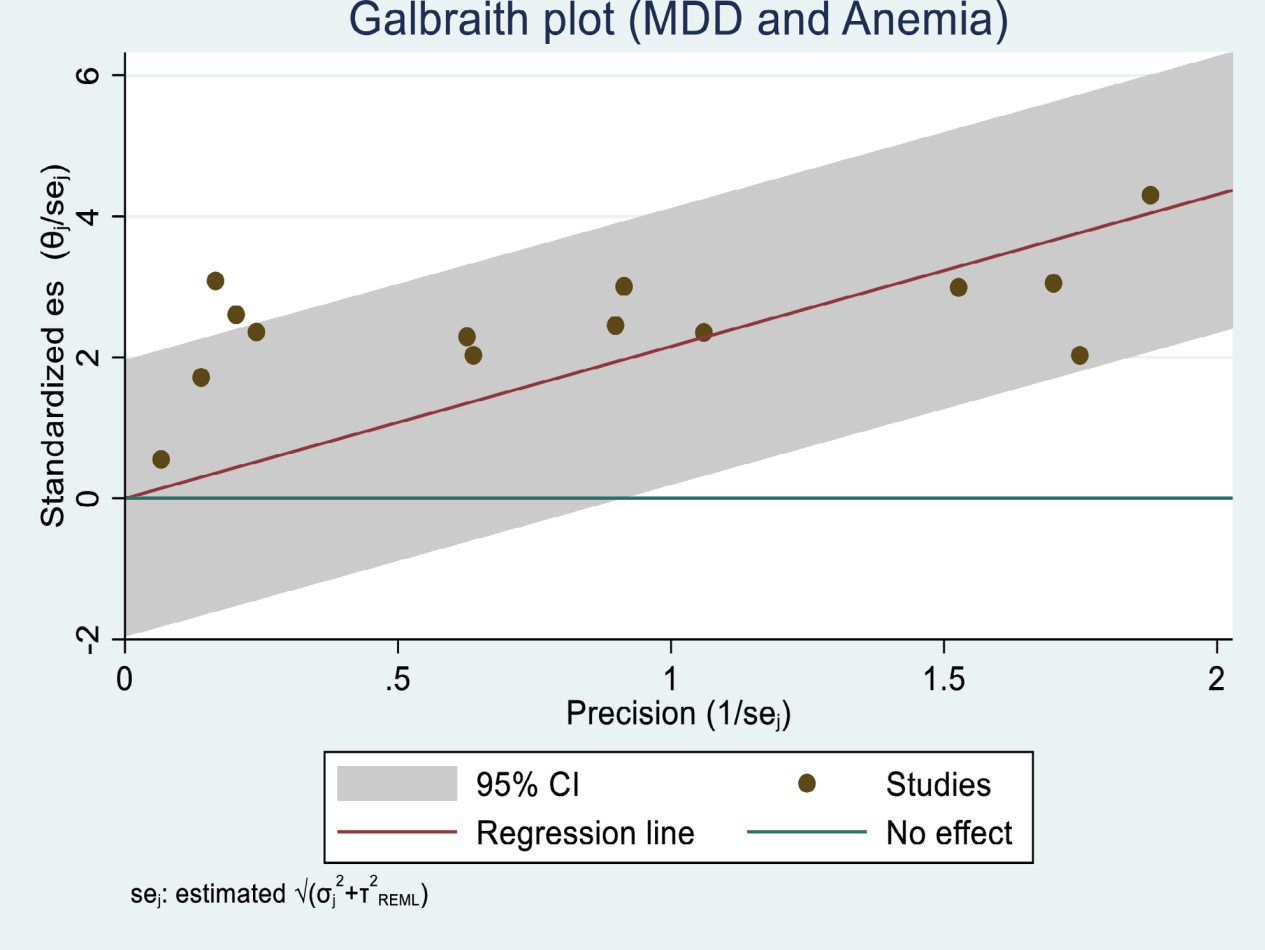
A Galbraith plot showing the association between dietary diversity during pregnancy and the risk of maternal anemia in Africa, 2022.

**FIGURE 3:**
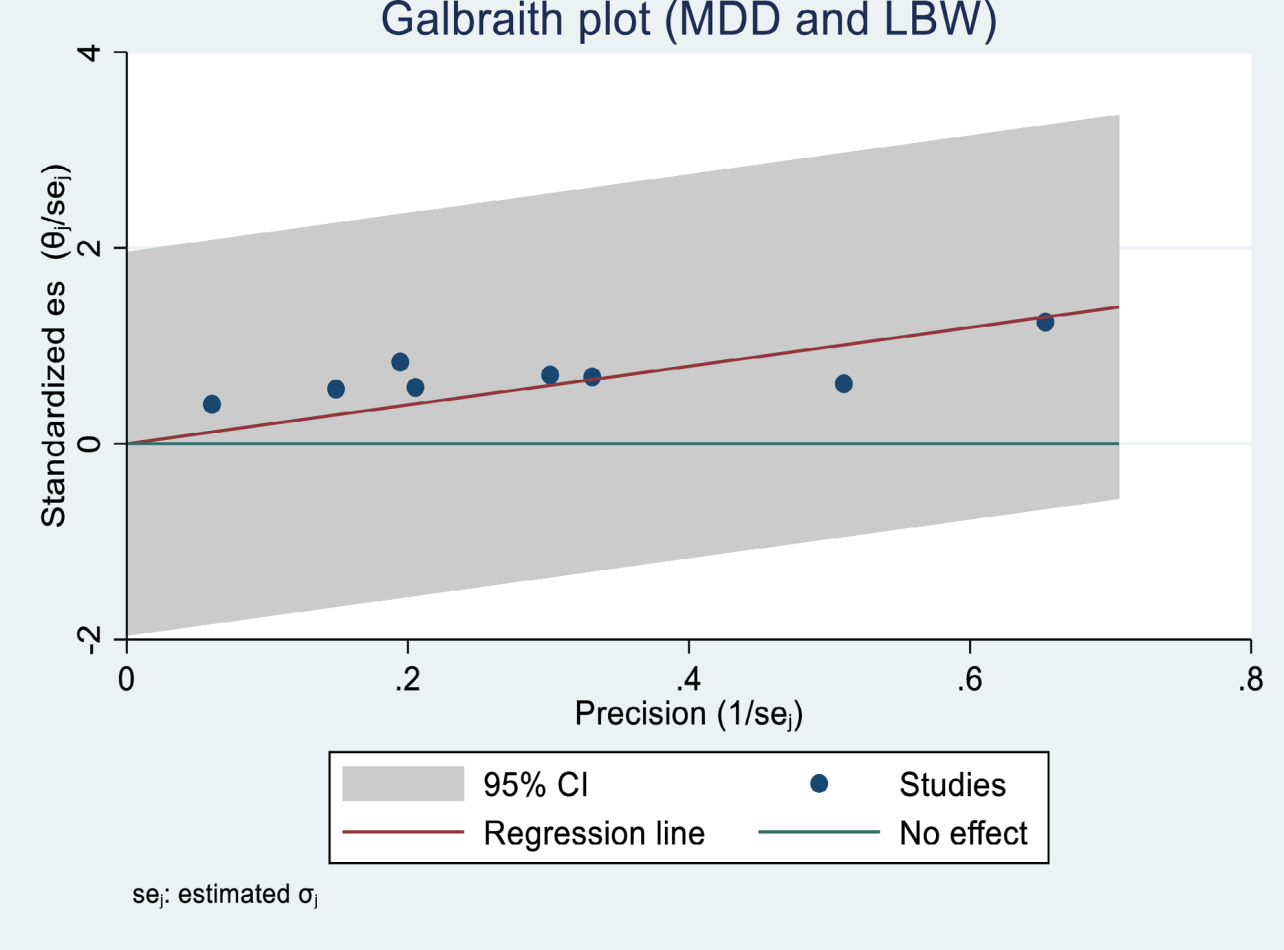
A Galbraith plot showing the association between dietary diversity during pregnancy and the risk of low birth weight in Africa, 2022.

Generally, studies included in the two outcome variables had low heterogeneity (<25%) (Higgins et al., 2003). The Chi‐square test for maternal anemia and low birth weight showed low heterogeneity among studies (*I* ^2^ = 14.79% and 9.97%, respectively). The variance among the reported studies was also small (*τ* ^2^ = 0.11 and 0.08), indicating that the prediction interval was also narrow. Similarly, the Galbraith plot also showed no heterogeneity; that is, no study rested on the “no effect line” (Figures 2 and 3).

### 3.5 Publication bias

**FIGURE 4:**
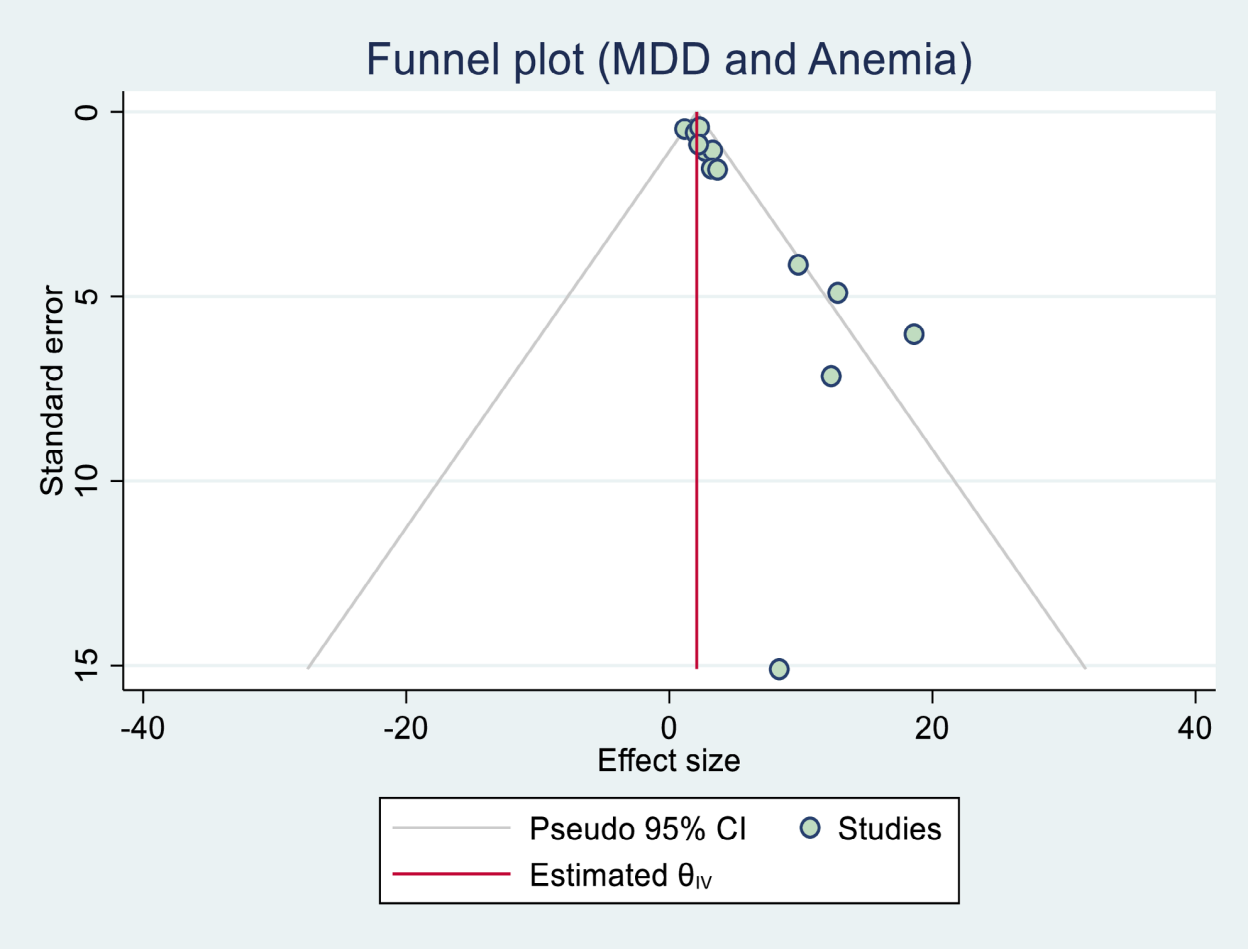
Funnel plot demonstrating publication bias for meta‐analysis of inadequate dietary diversity and risk of maternal anemia in Africa, 2022.

**FIGURE 5:**
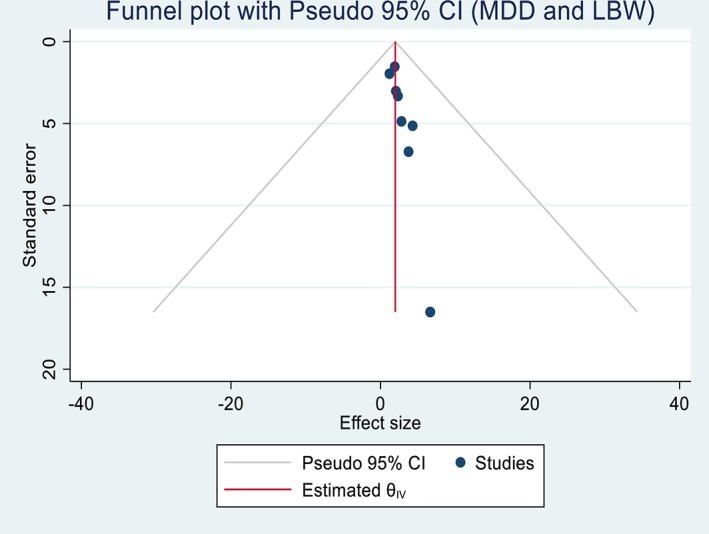
Funnel plot demonstrating publication bias for meta‐analysis of inadequate dietary diversity and risk of low birth weight in Africa, 2022.

**FIGURE 6:**
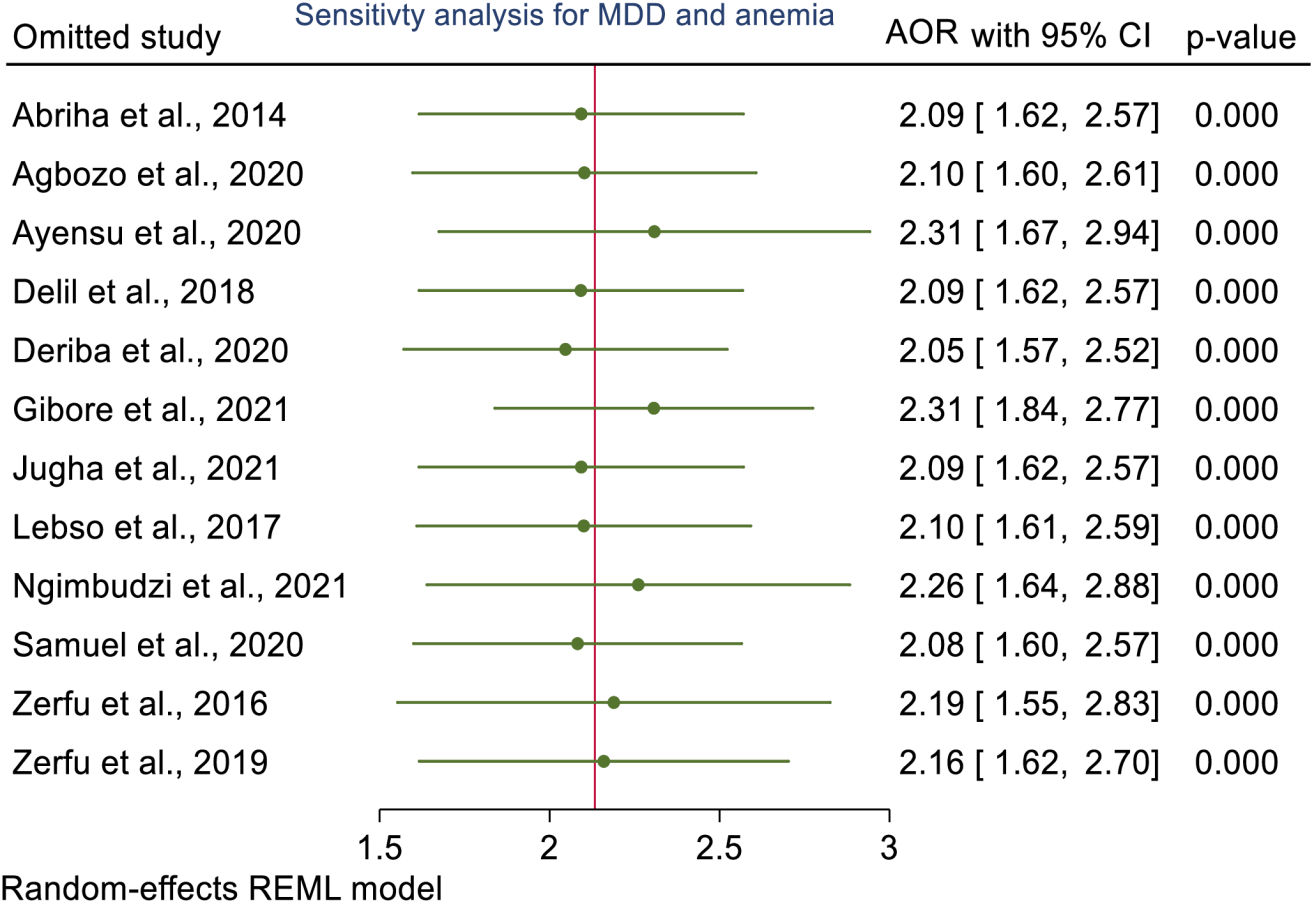
Sensitivity analysis for studies included in the meta‐analysis of dietary diversity during pregnancy and maternal anemia in Africa, 2022.

**FIGURE 7:**
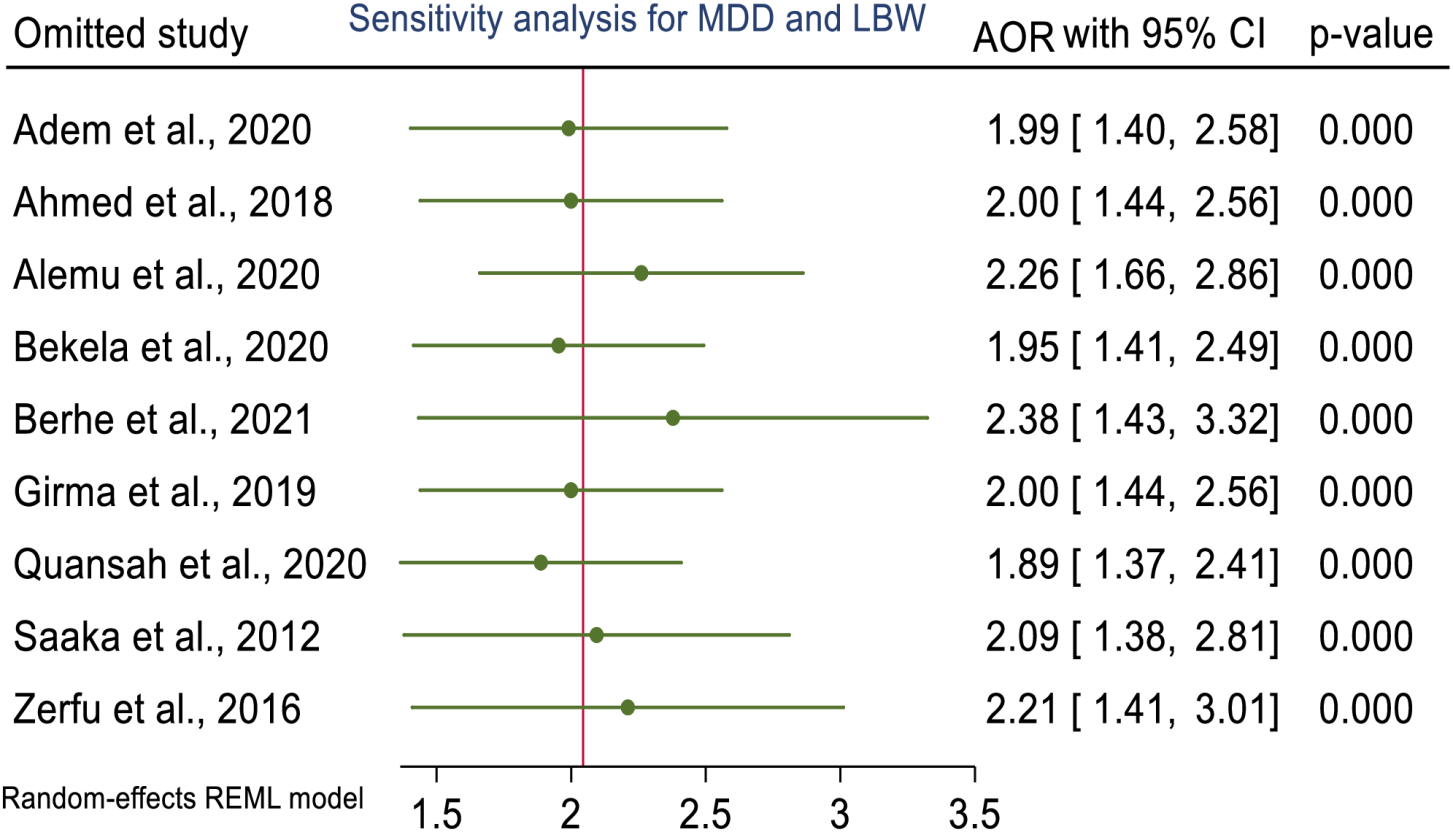
Sensitivity analysis for studies included in the meta‐analysis of dietary diversity during pregnancy and low birth weight in Africa, 2022.

Publication bias assessment between maternal dietary diversity during pregnancy and the risk of maternal anemia showed publication bias, as evidenced by funnel plots and Egger's test of 0.47. Similarly, the visual funnel plot and Egger's statistic test (0.39) of maternal dietary diversity and low birth weight showed potential for publication bias and a small study effect (Figures 4 and 5). In addition, sensitivity analyses showed that the overall results and conclusions were not influenced by differences in dietary assessment methods (24‐h recall versus FFQ). Therefore, the results of this study can be interpreted as having a high degree of certainty (Figures 6 and 7).

### 3.6 Inadequate dietary diversity during pregnancy and risk of maternal anemia

**FIGURE 8:**
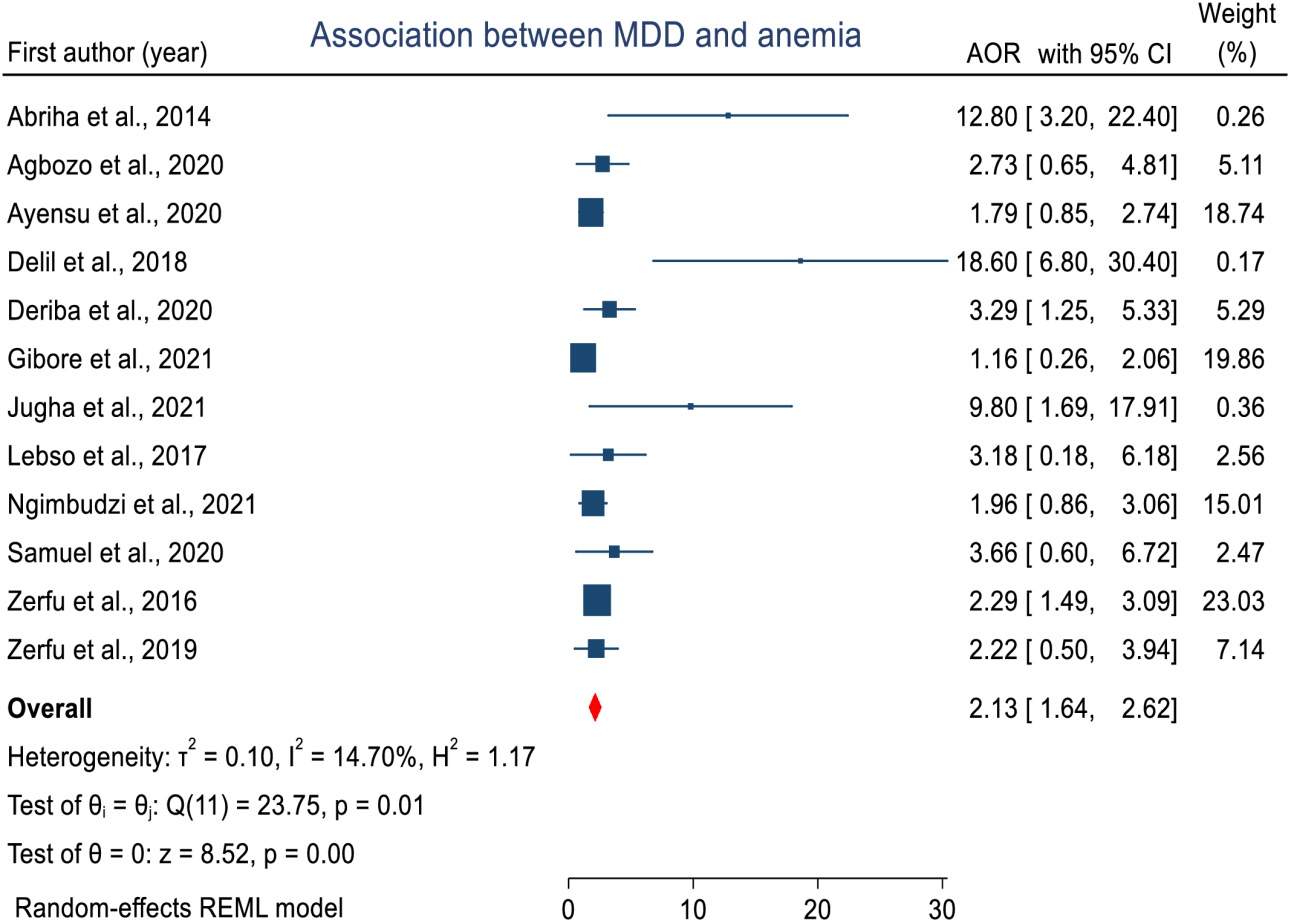
Forest plot that shows a pooled estimate of the association of dietary diversity during pregnancy and maternal anemia in Africa, 2022.

In this meta‐analysis, a random‐effects model was used to examine the association between inadequate dietary diversity during pregnancy and risk of maternal anemia. The pooled estimated adjusted odds ratio (AOR) was 2.15 (95% CI, 1.66–2.65) (Figure 8). Subgroup analysis by country showed that the pooled estimate of AOR was higher in Ethiopia (AOR = 2.6, 95% CI, 1.95–3.25); Ghana (AOR = 1.95, 95% CI, 1.09–2.81); and Tanzania (AOR =1.49, 95% CI, 0.72–2.27). Furthermore, subgroup analysis by study design showed that the highest estimate was found in case–control designs (AOR = 5.01, 95% CI, 1.93–11.95), followed by cohort designs (AOR = 2.33, 95% CI, 1.64–3.01), and the pooled estimate in cross‐sectional designed studies (AOR = 1.95, 95% CI, 1.27–2.63).

### 3.7 Inadequate dietary diversity during pregnancy and risk of low birth weight

**FIGURE 9:**
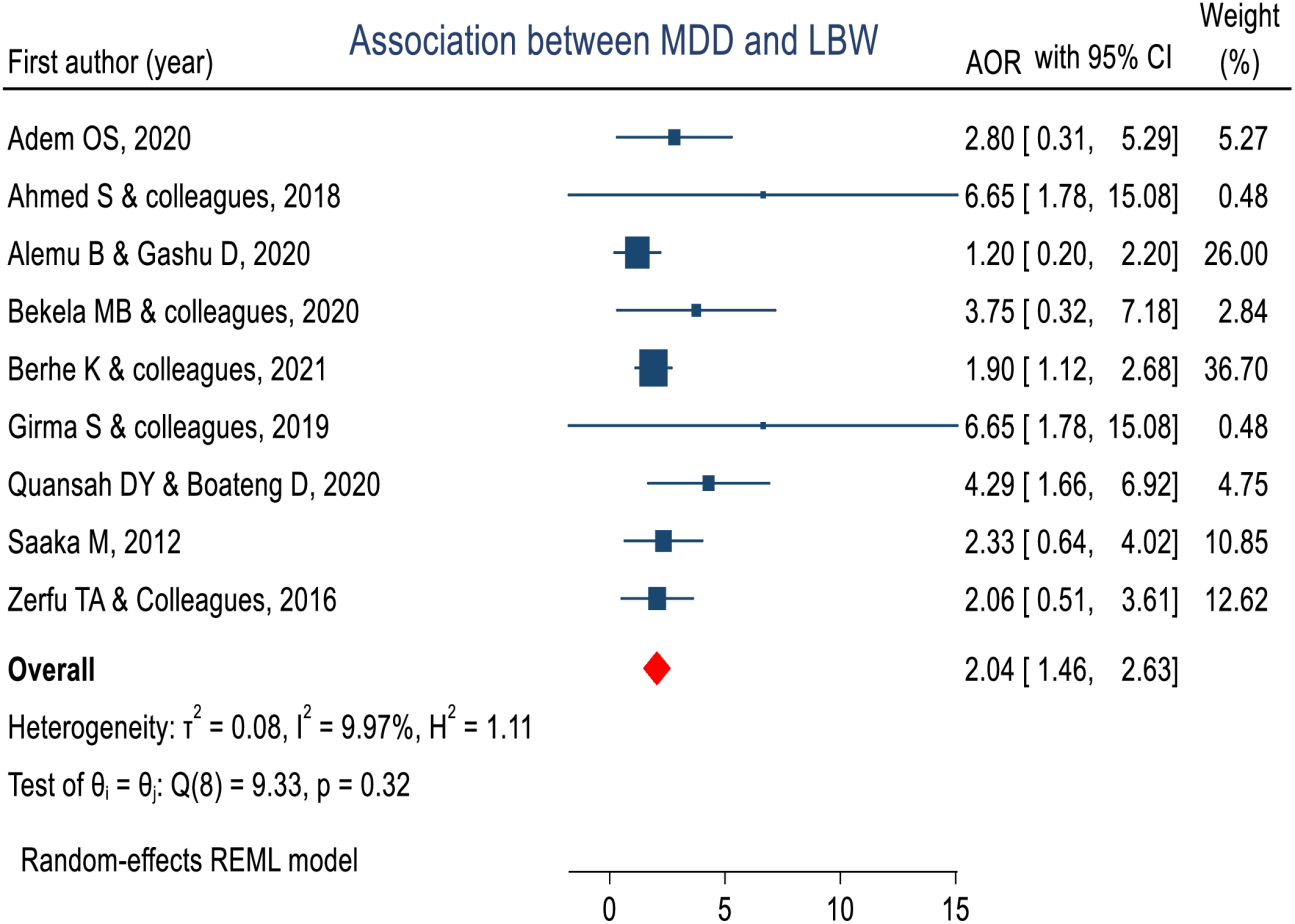
Forest plot that shows a pooled estimate of the association of dietary diversity during pregnancy and low birth weight in Africa, 2022.

We found nine eligible studies that reported an association between LBW and maternal dietary diversity during pregnancy. After the random‐effects meta‐analysis model was employed, the pooled estimated adjusted odds ratio was 2.04 (95% CI, 1.46–2.63) (Figure 9). Subgroup analysis by country showed that the pooled estimate in Ethiopia was 1.84 (95% CI, 1.29–2.39), and in Ghana was 3.04 (95% CI, 1.19–4.88). Similarly, subgroup analysis by study design showed pooled estimates in cross‐sectional studies (AOR = 3.04, 95% CI, 1.19–4.88), case–control (AOR = 3.49, 95% CI, 1.58–5.39), and cohort (AOR = 1.69, 95% CI, 1.12–2.26).

## 4 DISCUSSION

In Africa, a large percentage of pregnant mothers remain malnourished, and maternal nutrition and infant health are closely correlated. Maternal anemia and low birth weight remain major public health problems in developing countries (Zulfiqar et al., 2021). This study aimed to examine a pooled estimate of the association between dietary diversity during pregnancy, maternal anemia, and low birth weight in Africa.

Generally, dietary diversity optimizes micronutrient intake and improves pregnancy outcomes. According to this meta‐analysis, pregnant women who did not consume adequate dietary diversity had twice the odds of developing maternal anemia and giving a low birth weight compared to their counterparts. According to the FAO, women who achieve minimum dietary diversity are expected to have a higher likelihood of meeting their micronutrient intake recommendations (FAO & FANTA, 2016). Another study showed that MDD‐W not only indicates a higher intake of micronutrients but also greater consumption of fruits and vegetables, less consumption of red and processed meat, and sugar‐sweetened beverages (Gómez et al., 2020). However, the prevalence of inadequate dietary diversity in pregnant African women remains high. For example, in Ethiopia, a meta‐analysis showed that the prevalence of inadequate MDD‐W was 41%–53%. Food insecurity, large family size, rural residence, and a lack of knowledge about dietary diversity are also significantly associated with inadequate dietary diversity (Azene et al., 2021; Hidru et al., 2020). Furthermore, the results of this study were slightly lower than those of the meta‐analysis reported in Sub‐Saharan Africa (OR = 3.59). This might be attributable to widespread food insecurity in Sub‐Saharan Africa, which raises the risk of poor dietary diversity and maternal anemia in the region. People who are vulnerable to food insecurity have gone days without eating, increasing the risk of malnutrition (FAO, PAHO, WFP & UNICEF, 2019; Fraval et al., 2019). However, this finding is consistent with that of a meta‐analysis conducted in Ethiopia (Getaneh et al., 2021).

Furthermore, this study found that inadequate food diversity during pregnancy has a considerable impact on low birth weight. This finding is consistent with several studies (Gete et al., 2020; Karimi et al., 2022; Kibret et al., 2019) and a systematic review showed that a high intake of vegetables, fruits, whole grains, dairy products, and protein throughout pregnancy reduced the risk of small for gestational age (Gete et al., 2020). Dietary diversity is a good indicator of maternal nutrition and the likelihood of having a low‐birth infant, particularly in underdeveloped countries (Kheirouri & Alizadeh, 2021). The impact of maternal dietary diversity may extend to childhood stunting, as shown by a study on mothers of stunted children with a history of low consumption of pulses, dairy, eggs, and vitamin‐A‐rich fruits (Hasan et al., 2019). Similarly, adequate maternal dietary diversity is associated with higher child weight‐for‐height z‐scores (WHZ) and weight‐for‐age z‐scores (WAZ) and a lower risk of wasting (Huang et al., 2018). Moreover, an additional meta‐analysis determined a significant relationship between maternal anemia and low birth weight (Haider et al., 2013), indicating that the two outcome variables have a direct relationship (Figueiredo et al., 2018; Xiong et al., 2000).

Several studies have indicated that household food insecurity, low wealth index, rural residence, poor knowledge about nutrition, low educational status, and women living in large family sizes (>4) are more likely to have poor dietary diversity (Azene et al., 2021; Endalifer et al., 2021; Hidru et al., 2020; Moshi et al., 2022). This highlights the need to prioritize supportive programs for pregnant women with food insecurity who live in rural settings, have large families, or are uneducated. This also shows that women living in rural areas may have poor access to a variety of foods and inadequate nutrition knowledge.

Notably, UNICEF and Nutrition International are working to prevent all forms of malnutrition during pregnancy and breastfeeding (underweight, micronutrient deficiencies, and overweight) and to prevent low birth weight in newborns. One of UNICEF's programmatic priorities is to improve the coverage and quality of nutritional counseling before and during pregnancy and breastfeeding (UNICEF, 2021). Fortunately, ~10 countries in Africa have developed national food‐based dietary guidelines, and improving dietary diversity is a major component (Ainuson‐Quampah et al., 2022). Thus, the guideline helps provide maternal nutrition counseling given in the community and prenatal care clinics.

The strength of the study was enhanced and made conclusive by the use of meta‐analysis and lower heterogeneity within studies. However, the inability to access Scopus or Web of Science databases, the small number of included studies and countries, the limited number of comparable meta‐analysis studies, and the uneven cutoff points for defining inadequate dietary diversity among the studies were the limitations of this study. Despite this, the difficulty in accessing the Scopus and Web of Science databases was overcome by searching for published papers in large databases or search engines, such as Google Scholar.

## 5 CONCLUSION AND RECOMMENDATION

In Africa, inadequate dietary diversity during pregnancy doubles the odds of developing maternal anemia and low birth weight. Food insecurity, low wealth index, rural residence, and low educational status are the underlying factors associated with inadequate dietary diversity. Social protection programs that prioritize resolving food insecurity among pregnant women are imperative. Maternal nutrition promotion in the community as well as dietary counseling during antenatal care visits, preferably using culturally suited national food‐based dietary guidelines, should be strengthened. Furthermore, micronutrient supplementation and targeted fortification may be required in areas where food availability and accessibility are barriers. Further meta‐analyses that address additional factors associated with maternal anemia and low birth weight are recommended.

## AUTHOR CONTRIBUTIONS

**Awole Seid:** Conceptualization (lead); data curation (equal); formal analysis (equal); writing – original draft (lead); writing – review and editing (equal). **Desta Dugassa Fufa:** Data curation (supporting); investigation (supporting); software (lead); writing – original draft (supporting); writing – review and editing (supporting). **Misrak Weldeyohannes:** Conceptualization (supporting); resources (equal); writing – review and editing (equal). **Zuriyash Tadesse:** Methodology (equal); validation (equal); writing – original draft (equal). **Selamawit Lake Fenta:** Writing – review and editing (equal). **Zebenay Workneh Bitew:** Conceptualization (supporting); formal analysis (equal); writing – original draft (equal). **Getenet Dessie:** Formal analysis (equal); software (equal).

## FUNDING INFORMATION

The authors received no specific funding for this work.

## CONFLICT OF INTEREST STATEMENT

The authors declare that they have no competing interests.

## ETHICAL APPROVAL

Not applicable.

## CONSENT FOR PUBLICATION

Not applicable.

## Data Availability

All data generated or analyzed in this study are included in this published article.
